# The London School of Tropical Medicine

**Published:** 1901-03-30

**Authors:** 


### The Hospital

A Journal of

The Medical Sciences and Hospital Administration.

Vol. XXIX.—No. 757.] EDITED BY SIR HENRY BURDETT, K.C.B., AND SOLOMON C. SMITH, M.D., M.R.C.P. [March [illegible]

### The London School of Tropical Medicine.

Few things can be more interesting to any medical practitioner who desires to see a practical demonstration of some of the most important discoveries of recent times in reference to the arts of preventing and of healing disease than a visit to the small hospital near the Royal Albert Docks, to which is attached the London School of Tropical Medicine. By the liberal assistance of the Colonial Office and of a few private benefactors, a well lighted and efficient laboratory has been established, of which the chief, if not the only, fault is that it is already too small for the requirements of those who desire to use it. Attached to the laboratory is a house for the residence of students, who are expected to attend for a three months course' before they enter upon their duties as colonial surgeons, in the employment either of the Government, of missionary societies, or of commercial firms. The house contains a common room, dining-room, and six bedrooms for the students, who are thus spared the loss of time which would otherwise be incidental to daily journeying to one of the least accessible localities in the neighbourhood of town. The trains thither from Fenchurch Street, like those of the sometime "Great Eastern" as described by Thackeray in the early forties, certainly "must come in at last," but it cannot be denied that they occupy a considerable time in doing it. The Connaught Road Station once reached, however, the troubles of the traveller are over, and the detached addendum to the Seamen's Hospital is within a few yards of the line. Internally, it is exquisitely fitted up, with bright, airy, and well-lighted wards, which are maintained at a temperature suitable for patients, many of whom have but recently returned from hot climates. In the beds may be seen seamen of all nationalities; and there is scarcely a form of tropical disease which is not represented in its turn, the more serious of them frequently and constantly. On the occasion of a visit which we recently paid two men with Nigerian fever had but just been landed and put to bed, and in a few minutes the examination of a drop of blood from each revealed the presence of the parasite in the form of well-marked crescents. Another patient in the same ward afforded specimens of the amœba of dysentery. Signor Terzi, the Italian artist who accompanied Drs. Sambon and Low during their residence on the Campagna, near Ostia, in a mosquito-proof hut, is now engaged at the school as a portrait painter of mosquitoes and filariæ, and is producing a gallery of pictures of which it would be impossible to speak too highly, and of which absolute fidelity to nature is the chief characteristic. A cage of culices, replenished when necessary by special visits to their haunts, enables the students to acquire the power of dissecting these minute creatures in such a manner as to display their salivary glands and ducts, and to trace the progress of the ague organism to these ducts from the intestine. In fact, everything is provided which is calculated to equip a medical practitioner for arriving at a correct diagnosis of the various diseases produced by tropical parasites, and for conducting original investigations in quest of new light upon many of them.

Probably the most important work hitherto done in this direction has been the research into the causes of yellow fever recently undertaken by surgeons of the United States army in Cuba, and it is one that, so far, stands almost alone in the fact that the investigators were able to find soldiers who, with full knowledge of the risks involved, were nevertheless ready to submit themselves to the inoculation experiments which were essential to the attainment of a positive conclusion. In these men typical yellow fever was produced, in some by the bites of mosquitoes previously fed upon yellow fever patients, in others by the direct subcutaneous injection of the blood of such patients, with the remarkable difference that, while the blood acted almost immediately, the poison did not appear to reach the salivary glands of the insect, in such a way as to render its bite effective, until at least twelve days had elapsed since it was fed on the infected person. It will be remembered that, about the same time, Messrs. Durham and Myers were sent to Para, by the Liverpool Tropical School, to investigate the same disease, and that Mr. Myers crowned his devotion to duty by the sacrifice of his life. According to a brief "abstract" of a yet unpublished "interim report," it appears that Messrs. Durham and Myers discovered, in the tissues of bodies dead from yellow fever, a previously undescribed bacillus which they believed might be the cause of the malady, but they either were not able, or did not attempt, to bring this hypothesis to a practical test by inoculations with pure cultures. When the bacillus in question is again isolated, it will perhaps be found that others will be as ready as the American soldiers to risk their lives for the sake of humanity. The transmission of the fever by gnat-bites does not appear to support the notion of a bacillary origin; but in this, as in many other matters, we may still have much to learn, and there is ample scope for the work of as many investigators as can be brought into the field. The last portion of the American series of experiments, in which only negative results were obtained from the complete and prolonged exposure of unprotected persons to the sheets and garments which had been used and worn by yellow-fever patients, appears to show that the time-honoured fear of "fomites" may perhaps, before long, be consigned to the limbo of forgotten errors.

